# *In vivo* imaging of cerebral glucose metabolism informs on subacute to chronic post-stroke tissue status – A pilot study combining PET and deuterium metabolic imaging

**DOI:** 10.1177/0271678X221148970

**Published:** 2023-01-06

**Authors:** Anu E Meerwaldt, Milou Straathof, Wija Oosterveld, Caroline L van Heijningen, Mandy MT van Leent, Yohana C Toner, Jazz Munitz, Abraham JP Teunissen, Charlotte C Daemen, Annette van der Toorn, Gerard van Vliet, Geralda AF van Tilborg, Henk M De Feyter, Robin A de Graaf, Elly M Hol, Willem JM Mulder, Rick M Dijkhuizen

**Affiliations:** 1 Biomedical MR Imaging and Spectroscopy Group, Center for Image Sciences, University Medical Center Utrecht/Utrecht University, Utrecht, Netherlands; 2 BioMedical Engineering and Imaging Institute, Icahn School of Medicine at Mount Sinai, New York, USA; 3 Diagnostic, Molecular and Interventional Radiology, Icahn School of Medicine at Mount Sinai, New York, NY, USA; 4 Department of Internal Medicine and Radboud Center for Infectious Diseases, Radboud University Medical Center, Nijmegen, Netherlands; 5 Cardiovascular Research Institute, Icahn School of Medicine at Mount Sinai, New York, USA; 6 Icahn Genomics Institute, Icahn School of Medicine at Mount Sinai, New York, USA; 7 Department of Translational Neuroscience, University Medical Center Utrecht Brain Center, Utrecht University, Utrecht, The Netherlands; 8 Department of Radiology and Biomedical Imaging, Magnetic Resonance Research Center, Yale University School of Medicine, New Haven, CT, USA; 9 Department of Biomedical Engineering, Yale University School of Medicine, New Haven, CT, USA; 10 Department of Chemical Biology, Eindhoven University of Technology, Eindhoven, Netherlands

**Keywords:** Cerebral glucose metabolism, deuterium metabolic imaging, ischemic stroke, positron emission tomography, magnetic resonance imaging/spectroscopy

## Abstract

Recanalization therapy after acute ischemic stroke enables restoration of cerebral perfusion. However, a significant subset of patients has poor outcome, which may be caused by disruption of cerebral energy metabolism. To assess changes in glucose metabolism subacutely and chronically after recanalization, we applied two complementary imaging techniques, fluorodeoxyglucose (FDG) positron emission tomography (PET) and deuterium (^2^H) metabolic imaging (DMI), after 60-minute transient middle cerebral artery occlusion (tMCAO) in C57BL/6 mice. Glucose uptake, measured with FDG PET, was reduced at 48 hours after tMCAO and returned to baseline value after 11 days. DMI revealed effective glucose supply as well as elevated lactate production and reduced glutamate/glutamine synthesis in the lesion area at 48 hours post-tMCAO, of which the extent was dependent on stroke severity. A further decrease in oxidative metabolism was evident after 11 days. Immunohistochemistry revealed significant glial activation in and around the lesion, which may play a role in the observed metabolic profiles. Our findings indicate that imaging (altered) active glucose metabolism in and around reperfused stroke lesions can provide substantial information on (secondary) pathophysiological changes in post-ischemic brain tissue.

## Introduction

Ischemic stroke induces acute disruption of cerebral energy metabolism that may sustain for days to weeks,^1,2^ even after reperfusion.^3^ Prevalent treatment of acute ischemic stroke relies on either intravenous thrombolysis or intra-arterial thrombectomy for recanalization.^4^ After its success in clinical randomised controlled trials, endovascular recanalization has become the preferred intervention for patients with acute large vessel occlusions.^5,6^ Despite this breakthrough, a subset of patients still has a relatively poor outcome after reperfusion treatment.^7^ This may be attributed to several factors including time-to-treatment,^8^ occlusion location, and ischemic core size.^9^ Hence, restoration of perfusion by itself may not be sufficient for improved stroke outcome. A mismatch between perfusion and cerebral metabolism lasting beyond the 24 hour time window can influence stroke outcome.^10^ Therefore, a relatively poor outcome after successful reperfusion therapy could be (partially) linked to inefficient or altered glucose metabolism, and monitoring (alterations in) cerebral metabolism may potentially improve patient care.

Beyond stroke-induced changes in cerebral blood flow, oxygen extraction fraction and cerebral metabolic rate of oxygen, information on post-stroke glucose metabolism and its effects on stroke outcome remain scarce. Elevated lactate levels, indicative of anaerobic glycolysis as a result of cerebral ischemia, can persist after reperfusion, as shown in experimental stroke models.^11^ In human stroke patients, lesion severity-dependent cerebral lactate accumulation has been shown to return to normal levels within 2 to 4 weeks.^12,13^ Importantly, these studies quantified lactate by proton (^1^H) magnetic resonance spectroscopy (MRS), which cannot differentiate between actively produced or passively present lactate in the post-stroke brain. Injection or infusion of glucose labelled with stable or radioactive isotopes allows detection of glucose uptake and its metabolites, from which active glucose metabolism can be measured. Studies assessing active glucose metabolism in the post-stroke brain beyond the 24 hour time window have been scarce. A previous study by our group showed active formation of lactate, glutamate and glutamine in and around the lesion 3 weeks after 90 min transient middle cerebral artery occlusion (tMCAO) in rats, which coincided with functional recovery.^3^

In addition to neuronal changes, stroke triggers an inflammatory cascade, which can continue for several days to weeks after stroke^14^ and may explain persistent metabolic activity in the lesion territory despite neuronal death. Additionally, stroke has been associated with impaired cerebral clearance mechanisms,^15,16^ potentially affecting the accumulation of glucose and its metabolites.

Cerebral glucose metabolism has been previously evaluated in experimental animals based on *post mortem* bioluminescence and autoradiography analyses, as well as *in vivo* with translational positron emission tomography (PET) and magnetic resonance imaging (MRI) methods.^17^ MRI- and PET-based methods, such as (^1^H/)^13^C MRS, GlucoCEST, [^18^F]fluorodeoxyglucose (FDG) PET and deuterium (^2^H) metabolic imaging (DMI),^18^ enable imaging of active glucose metabolism in the brain. The latter two approaches may be considered the most applicable in clinical settings.^19,20^ FDG PET, which is widely adopted in the clinic, allows assessing cellular glucose uptake, but does not inform on downstream glycolysis. DMI, a new MR-based imaging technique,^18,21,22^ on the other hand, is capable of spatially mapping downstream metabolites of glucose. Combined, these methods may provide a comprehensive picture of active glucose metabolism after ischemic stroke.

In the current study, we applied clinically applicable imaging methods to assess cerebral glucose metabolism beyond the initial 24 hours after ischemia-reperfusion in order to obtain information on secondary (patho)physiological events that could influence stroke outcome. We hypothesised that the cerebral glycolytic profile may differentiate between mild-to-moderate and severe stroke injury, and that ongoing glucose metabolism in and around a stroke lesion has different cellular sources. In addition we measured changes in metabolite levels after glucose administration for evaluation of possible disturbance in cerebral clearance systems.

## Materials and methods

### Animals

All animal experiments were approved by and performed in accordance with regulations from the Icahn School of Medicine at Mount Sinai Institutional Animal Care and Use Committee (IACUC), and the Animal Experiments Committee of the University Medical Center Utrecht and the Utrecht University. Experiments were performed in accordance with the guidelines of the European Communities Council Directive (2010/63/EU). The study was performed with adult (11–16 weeks old) male C57BL/6 mice (Charles River, Germany (DMI and immunohistochemistry) and Jackson Laboratories, USA (FDG PET)). Animals were housed under standard conditions with a light/dark cycle of 12/12 hours and *ad libitum* access to food and water. Animal data are reported in accordance with the ARRIVE 2.0 guidelines.^23^ Mice were randomly assigned to control or subacute/chronic post-stroke groups.

### Stroke model

Stroke was induced in the right cerebral hemisphere by a transient occlusion of the middle cerebral artery (tMCAO)^24^ under isoflurane anaesthesia (4% for induction, 1.5–2% (in O_2_) for maintenance). Body temperature was maintained at 37°C by a heated pad. The common, external, and internal carotid arteries were dissected. A 230 µm thick silicon-coated filament (#602345PK10Re, Doccol, USA) was intraluminally advanced into the internal carotid artery via the external carotid artery until resistance was felt at the MCA (middle cerebral artery) bifurcation. The filament was withdrawn after 60 minutes, after which the animals were allowed to recover from anesthesia. 60-minute tMCAO in mice models moderate to severe stroke, with consistent ischemic damage in the striatum and variable injury in cortical areas.^25^ Control mice did not undergo any procedures prior to the imaging experiments.

### MicroPET/CT acquisition

At 48 hours or 11 days after stroke, mice (n = 15) were injected with 2-deoxy-2-[^18^F]fluoro-D-glucose (302.4 ± 16.4 µCi) in phosphate-buffered saline (PBS) through a tail vein catheter. The animals were kept under isoflurane anaesthesia (4% for induction, 1.5–2% (in O_2_) for maintenance) and the radiotracer was allowed to circulate for 60 minutes prior to a static PET/CT scan with a nanoScan PET/CT scanner (Mediso, Hungary). A 3-min whole-body CT scan (energy 50 kVp, current 180 µAs, isotropic voxel size 0.25 mm^3^) was followed by a 20- or 30-min static PET scan. Coincidences were filtered with an energy window between 400 and 600 keV. Reconstruction was performed with four full iterations, six subsets per iteration with an isotropic voxel size of 0.4 mm^3^ using the TeraTomo 3D reconstruction algorithm (Mediso Nucline nanoScan v 3.00.020.0000). CT-based attenuation correction was applied for PET reconstruction. Animals were euthanized with an overdose of isoflurane immediately after the PET/CT scan.

### PET/CT analyses

Image analyses were performed with Osirix MD v.10.0.4 (Pixmeo SARL, Switzerland) by fusing the whole-body CT images with the PET images. Circular regions-of-interest (ROIs) inside the MCA territory of each brain hemisphere were drawn over several coronal slices from 0 to about 3 mm posterior to bregma. Similar regions were drawn for stroke and control animals. The mean standardised uptake value (SUV, g/mL) was calculated for the ipsi- and contralateral hemispheres using the ROIs, back-propagated to the PET images.

### MR acquisition

C57BL/6 mice (n = 26) were anaesthetised with isoflurane (4% for induction, 1.5–2% (in O_2_) for maintenance) 48 hours or 11 days after stroke. A tail vein catheter (SAI #PU-025-50; 27 G needle) filled with saline (Bauer) was inserted and the animal was placed in the scanner. Data was acquired using a 9.4 T MR system equipped with 400 mT/m gradient coil (Agilent). Anatomical images were acquired with a home-built 90 mm diameter Helmholtz ^1^H volume coil, and metabolic images with a home-built curved 12 mm (r = 7.5 mm) ^2^H surface coil. A T2-weighted anatomical image of the brain was acquired using fast spin echo multi-slice acquisition (repetition time (TR) = 2363.6 ms, echo spacing = 14 ms, segments/echo train length = 16/8, 16 averages, 20 consecutive 0.7 mm slices, field of view = 25 × 25 mm^2^, matrix size = 128 × 128) in 10 minutes, followed by a 3D gradient echo (GE) acquisition (TR = 4.05 ms, echo time = 2.04 ms, flip angle = 20°, field of view = 25 × 25 × 25 mm^3^, matrix size = 99 × 99 × 99). ^2^H MR imaging consisted of steady-state and dynamic DMI. Steady-state images of the brain were acquired with a 3D ^2^H MR spectroscopic imaging (MRSI) matrix of 11 × 11 × 11 with 2.5 ×2.5 × 2.5 mm^3^ (∼16 µl) nominal spatial resolution in approximately 36 minutes (TR = 400 ms, 8 averages). The same field of view was applied as for the anatomical 3D GE acquisition (for metabolite localisation). Signal excitation was achieved with an adiabatic pulse of 2000 µsec at 55 W. Steady-state scans were acquired once before and again 90 minutes after the start of infusion of deuterium-labelled glucose (D-glucose-6,6-d2, Buchem BV, Apeldoorn, the Netherlands).The glucose was dissolved in filtered demi-water to a 1M concentration. After 125 minutes of glucose infusion (variable speed, 1.95 g/kg), a dynamic clearance measurement was executed with an adapted Image Selected In vivo Spectroscopy (ISIS) sequence^26^ where only slice selective editing in sagittal direction was used, distinguishing between the two hemispheres (TR = 500 ms, TI = 4 ms, 300 averages, acquisition time approx. 7.5 minutes), which was repeated ten times.

### MR processing and quantification

#### Baseline and steady-state DMI

^1^H MRI and DMI data were processed using FSL tools^27^ and NMRWizard (a custom-made graphical user interface in MATLAB 9.5). DMI processing of the metabolite levels was done using a 11 × 11 × 11 MRSI grid. It included 5-Hz line broadening followed by 4D Fourier transformation and spectral integration. MRSI data were thresholded based on the quality of the scan to include voxels with sufficient signal-to-noise ratio (SNR) in the subsequent analyses. The spectra were fitted using linear least-squares fitting of up to four Lorentzian lines and a linear baseline. Because the chemical shifts of deuterium-labelled metabolites are highly reproducible and insensitive to the chemical environment, we linked the frequencies of the metabolites to the frequency of water, with a maximum frequency shift per metabolite of 5 Hz. The fit results were manually checked. As a basis for the fitting the relative frequencies of glutamate/glutamine, glucose and lactate to water were used with a maximum line width of 30 Hz. The relative frequencies were derived by quantifying the average relative frequencies from the global spectra of two randomly selected datasets.

In order to extract concentrations of the metabolites, the metabolite maps were corrected for incomplete T1 relaxation, label loss in the glycolysis and TCA cycle^18^ and number of deuterons per metabolite. Concentrations were estimated based on baseline water concentration. To correct for incomplete T1 relaxation the maps were divided by the T1 saturation values^18^ (water: 320 ms; glucose: 64 ms; glutamate/glutamine: 146 ms; lactate: 297 ms). Correction for label loss and for the number of deuterons was performed by dividing the metabolite maps with the number of deuterons and label loss for each metabolite, as described by De Feyter et al.^18^ Finally, the steady-state metabolite maps were divided by the natural abundance water peak where the water concentration was assumed to be 10.12 mM (natural abundance of ^2^H in water). These corrected, non-interpolated metabolic maps were used for all quantifications. In order to quantify metabolite concentrations in the brain, the metabolic maps were resampled to match the anatomical images with resampled voxels in the 2.5 × 2.5 × 2.5 mm^3^ area containing the same value as the original MRSI voxel. For visualisations the metabolic maps were converted to 100 × 100 × 100 voxel maps using sinc interpolation (MRTrix v 3.0).

#### Dynamic DMI

Dynamic metabolite clearance data consisted of three spectra from the whole brain, left and right hemispheres, respectively, for all acquired time points. The spectra were phase- and frequency-corrected, and the water peak alignment was manually checked. After Fourier transformation, spectra were fitted, and similar procedures were performed to extract metabolite concentrations as described above. In order to compare the clearance of metabolites across animals, all dynamically measured metabolite concentrations were normalised to values obtained from the first dynamic measurement after stopping the deuterated glucose infusion.

#### Lesion masks

Stroke lesions were manually outlined on the T2-weighted images. Lesion masks were projected to the anatomical 3D GE images using FMRIB's Linear Image Registration Tool (FLIRT)^28^ and FNIRT. Lesion masks were used as an inclusion area for quantification of the metabolites within the ipsilateral hemisphere (i.e. the lesion core). Additionally, a mirrored homologue of the lesion masks was projected to the contralateral hemisphere and used for quantifying metabolites within the contralateral hemisphere. Lesions were categorized as mild/moderate or severe based on lesion extent, where mild/moderate lesions involved only subcortical areas and severe lesions involved subcortical and cortical areas. For metabolite quantification in control animals and in cortical areas of animals with mild/moderate lesions, lesion incidence maps were used. The incidence maps were generated by projecting lesion masks from mice with severe lesion at 48 hours to a reference space (using FLIRT and FNIRT) and calculating lesion incidence on a voxel-basis. The resulting lesion incidence map was thresholded to include voxels with a lesion incidence of minimally two mice, after which it was binarized to obtain a lesion mask. The lesion mask was registered to images of individual animals and metabolite quantifications were performed in this area, similarly to mice with severe stroke. Lesion mask voxels extending to MRSI voxels with insufficient SNR were excluded.

### Immunohistochemistry

Mice from a separate group (n = 4) were sacrificed and transcardially perfused with cold saline followed with 4% paraformaldehyde in PBS. For each experimental group tissue of one mouse was used for immunohistochemistry. The brains were extracted and post-fixated in 4% paraformaldehyde in PBS at 4°C overnight, after which they were stored in 30% sucrose in PBS. The brains were frozen and sectioned in slices of 20 µm thickness, which were mounted on microscope slides and stored at −20°C until staining.

The slides were defrosted at room temperature and washed three times with PBS for 10 minutes. Next, the slides were incubated in PBS-BT (PBS, 3% BSA, 1% Triton X-100) for 30 minutes. The brain sections were then incubated with primary antibodies diluted in PBS-BT overnight at 4°C. Each section was incubated with rabbit anti-GLUT1 (glucose transporter 1, 1:5000, 07-1401 Sigma-Aldrich) and one of the following antibodies: mouse anti-GFAP (glial fibrillary acidic protein, 1:500, G3893 Sigma-Aldrich), goat anti-Iba1 (ionised calcium-binding adapter molecule 1, 1:750, ab5076 Abcam) or mouse anti-NeuN (neuronal nuclear protein, 1:800, MAB377 Sigma-Aldrich).

The next day, the sections were washed in PBS (3 × 10 minutes) followed by a one hour incubation with the secondary antibodies in PBS-BT (Alexa 488-conjugated donkey anti-mouse, A21202 Invitrogen; Alexa 488-conjugated donkey anti-goat, A32814 Invitrogen; Alexa 594-conjugated donkey anti-rabbit, A21207 Invitrogen) in the dark at room temperature. The sections were washed in PBS for 3 × 10 minutes and sealed with FluoroShield containing DAPI (F6057 Sigma-Aldrich). The slides were stored at 4°C until imaging with a wide field fluorescence microscope (Zeiss Axio Scope A1). The contrast and brightness of the images with 20x magnification was adjusted in ImageJ (v. 1.52k).

### Statistical analyses

Statistical analyses were performed with GraphPad Prism (v. 9.3.1). A nonparametric Kruskall-Wallis test was used for the analyses befitting small sample sizes except for the dynamic data where a linear mixed model was used. Dunn’s test was used for corrections for multiple comparisons. Two-way ANOVA with Šidák correction was used to test differences in metabolite levels between ipsi- and contralateral ROIs. Because of the exploratory nature of our study, focusing on evaluation of the potential of DMI to assess glucose metabolism in post-stroke mouse brain, we used relatively small sample sizes. Mice were excluded when no lesion was detected on T2-weighted MRI scans. DMI data were excluded when the glucose signal was absent in the brain, indicative of defective intravenous infusion. The experimenter who analysed the imaging data was blinded to the post-stroke time point.

## Results

For FDG PET experiments a total of fifteen mice were used. For DMI, a total of 26 mice was used, of which ten mice were excluded because of absence of a lesion in the anatomical MR images (n = 3), death before the post-stroke imaging end-point of 11 days (n = 6), or failed glucose infusion (n = 1). Hemorrhagic transformations or hematomas were not detected in the anatomical MR images. Four mice were used for immunohistochemistry.

### Transiently reduced glucose uptake after stroke

**Figure 1.**
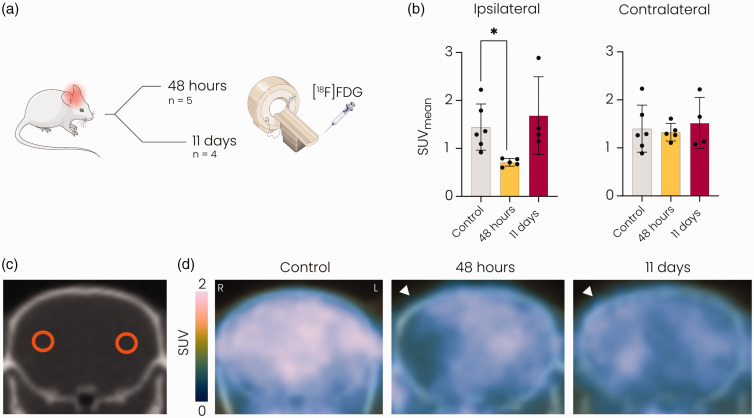
[^18^F]FDG PET revealed reduced glucose uptake at 48 hours but not at 11 days after stroke in the ipsilateral MCA territory. (a) Overview of the experiment. Mice underwent [^18^F]FDG] PET either 48 hours or 11 days after 60-minute tMCAO. (b) Mean standard uptake value (SUV) was significantly decreased in the ipsilateral hemisphere at 48 hours compared to the control group and normalised at 11 days after stroke. (c) Representative CT image showing circular ROIs in the ipsi- and contralateral MCA territories and (d) Representative [^18^F]FDG] PET images overlaid on CT images of the brain showing reduced glucose uptake in the MCA territory of the ipsilateral hemisphere (white arrowhead) at 48 hours but not at 11 days after tMCAO. SUV: standard uptake value; MCA: middle cerebral artery. *p < 0.05 vs. control with Kruskall-Wallis test. n = 6 for controls, n = 5 for 48 hours post-stroke and n = 4 for 11 days post-stroke mean ± SD.

In vivo [^18^F]FDG] PET/CT was performed to assess glucose uptake in the brain at 48 hours and 11 days after stroke (Figure 1(a)). Quantification of the mean [^18^F]FDG signal in the brain showed reduced glucose uptake (Figure 1(b)–(d)) in the ipsilateral MCA territory at 48 hours but not at 11 days after tMCAO, compared to control mice (*p* < 0.05). No changes were observed in glucose uptake in the contralateral MCA territory after stroke.

### DMI reveals time-dependent changes in active glucose metabolism after stroke

**Figure 2.**
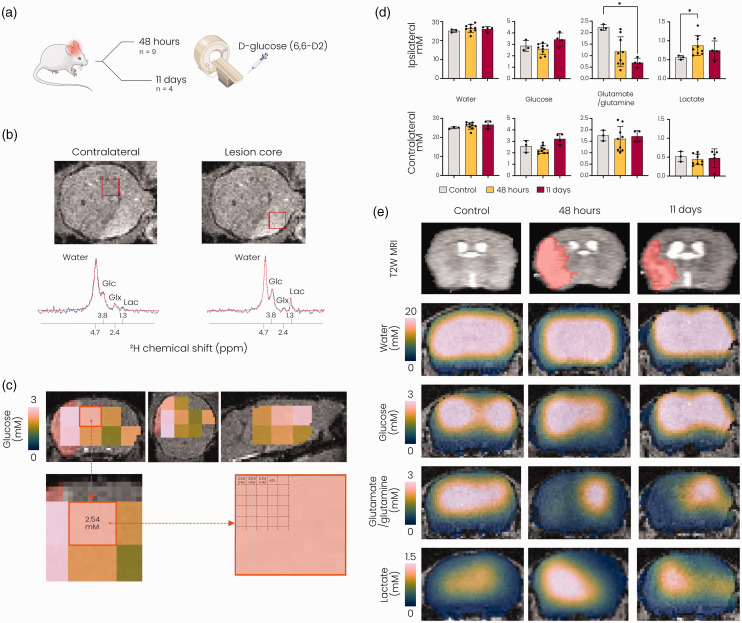
DMI reveals changes in active glucose metabolism after stroke. (a) Overview of the experiment. Mice underwent DMI either 48 hours or 11 days after 60-minute tMCAO. (b) Top: T2-weighted MR image of an axial mouse brain slice 48 hours after tMCAO, displaying the lesion as a hyperintense region. Bottom: Fitted (*red*) and original (*blue*) spectra obtained from voxels (shown in red in the T2-weighted images) in the contralateral hemisphere (*left*) and the lesion core (*right*) 48 hours after tMCAO. (c) MRSI voxels with sufficient SNR for further metabolite analysis overlaid on anatomical T2-weighted MRI images in coronal (*left*), axial (*middle*) and sagittal (*right*) views (data from a representative mouse). Lesion mask shown in red under the MRSI voxels. The resolution of the MRSI was artificially increased to match the underlying anatomical image. (d) Metabolite concentrations (mM) quantified in lesioned tissue and in its contralateral counterparts. Metabolite values in the control brain were derived from ROIs based on lesion incidence maps at 48 hours after stroke and (e) Representative T2-weighted MR images (top row) and interpolated metabolite concentration maps overlaid on T2-weighted MRI images (bottom rows) for a control mouse and mice at 48 hours and 11 days after stroke. Due to the removal of voxels with low SNR in the perimeter of the brain, interpolation led to erroneous display of lower concentrations in the brain boundaries. SNR: signal-to-noise ratio; Glc: glucose; Glx: glutamate/glutamine; Lac: lactate; MRSI: magnetic resonance spectroscopic imaging, *p < 0.05 vs. control with Kruskall-Wallis test. n = 3 for controls, n = 9 for 48 hours post-stroke and n = 4 for 11 days post-stroke, mean ± SD.

We measured active glucose metabolism in post-stroke mouse brain at 48 hours and 11 days after stroke using ^1^H/^2^H MRI with deuterium-labelled glucose (D-glucose-6,6-d2) as a substrate (Figure 2(a)). Individual spectra obtained from the lesion core showed emergence of lactate and repression of glutamate/glutamine formation at 48 hours after stroke (Figure 2(b)). Quantification of metabolite concentrations in the lesion core and its contralateral counterpart (excluding voxels partially outside of the brain or with insufficient signal-to-noise ratio (Figure 2(c))), revealed altered glucose metabolism at 48 hours and 11 days after stroke (Figure 2(d)). While the concentration of deuterated glucose and deuterated water in the brain after intravenous infusion was unaltered, glutamate/glutamine production was significantly reduced in lesioned tissue at 11 days after stroke (*p < *0.05). Increased lactate formation was measured at 48 hours (*p < *0.05), but not at 11 days after stroke. Representative interpolated metabolic maps illustrate the spatial distribution of the measured glucose metabolism products after stroke (Figure 2(e)).

### Metabolic fingerprint subacutely after stroke depends on lesion severity

**Figure 3.**
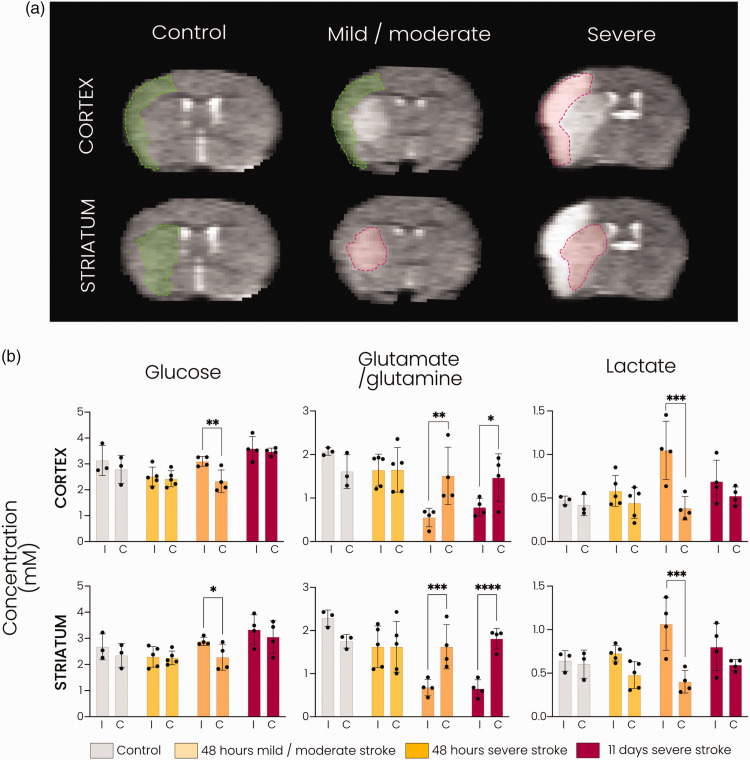
DMI reveals distinct metabolic fingerprints associated with lesion severity subacutely after stroke. (a) Representative T2-weighted MR images of coronal mouse brain slices showing cortical and striatal ROIs. The ROIs in green (cortical and striatal for the control group; cortical for the mild/moderate stroke group) are based on lesion incidence maps from severe stroke animals at 48 hours. The ROIs in red (striatal for the mild/moderate stroke group; cortical and striatal for the severe stroke group) are based on lesioned tissue in the individual animal at 48 hours or 11 days after tMCAO and (b) Metabolite concentrations (mM) in the cortical and striatal ROIs in controls and at 48 hours and 11 days after stroke. *p < 0.05, **p < 0.01, ***p < 0.001, ****p < 0.0001 with two-way ANOVA with Šidák correction. n = 3 for control, n = 5 for mild/moderate stroke, n = 4 for severe stroke at 48 hours and n = 4 for severe stroke at 11 days, mean ± SD.

Sixty minute tMCAO resulted in different lesion sizes, involving subcortical areas, i.e. striatum, only (mild/moderate stroke group: n = 5 for 48 hours group; n = 0 for 11 days group) or subcortical and cortical areas (severe stroke group: n = 4 for 48 hours; n = 4 for 11 days). To investigate the influence of lesion severity on glucose metabolism in the subacute phase, i.e. 48 hours post-stroke, we stratified mice based on lesion type (Figure 3(a)). We selected the injured subcortical (i.e. striatal) and cortical areas as ROIs for metabolite quantifications in stroke animals (Figure 3(a)). For control animals and for the cortical areas in animals with mild/moderate stroke, the ROIs were based on the lesion incidence map of the severe stroke group.

In the lesioned cortical and striatal tissue of animals with severe stroke lesions, glutamate/glutamine formation was significantly lowered compared to the contralateral hemisphere at 48 hours (p < 0.01 and p < 0.001, respectively) and at 11 days (p < 0.05 and p < 0.0001, respectively). In animals with mild/moderate stroke lesions, glutamate/glutamine labelling was largely preserved in the lesioned striatal tissue as well as in the non-lesioned ipsilateral cortical tissue (Figure 3(b), middle). Lactate production was significantly elevated in cortical and striatal areas of severe lesions (p < 0.001 vs. contralateral) at 48 hours post-stroke, while no significant increase was measured in the striatal or bordering cortical area of mild/moderate lesions or within severe lesions at 11 days post-stroke (Figure 3(b), right). The level of deuterated glucose was significantly higher in the ipsilateral cortex and striatum in the severe stroke group (p < 0.01 and p < 0.05 vs. contralateral, respectively) at 48 hours post-stroke, whereas no difference was observed in the controls or other stroke groups (Figure 3(b), left).

### Dynamic DMI enables measurement of metabolite clearance

**Figure 4.**
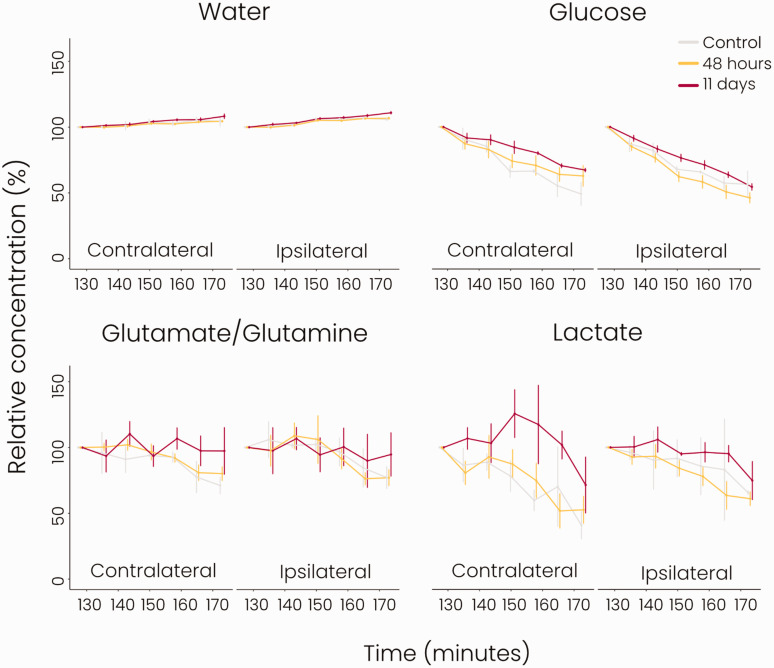
Clearance of glucose and its metabolites measured with dynamic DMI after glucose infusion (*grey = control group, yellow = 48 hours post-stroke, red = 11 days post-stroke*). Concentrations (%) were normalised to the first post-infusion dynamic measurement (Supplemental Figure 1). There were no significant differences in the clearance profiles in the ipsi- or contralateral hemispheres between groups. *n = 2 for control, n = 9 for 48 hours post-stroke, n = 4 for 11 days post-stroke, mean ± SD.*

After 125 min of deuterated glucose infusion, we performed dynamic DMI to measure the clearance of remaining glucose and the actively formed metabolites. Results are shown in Figure 4. About half of the deuterated glucose in the brain was washed out in three hours. A similar clearance rate was observed for lactate in control animals and animals at 48 hours post-stroke, while a trend of slower lactate clearance was apparent at 11 days after stroke. Deuterated glutamate/glutamine levels also declined but remained higher than glucose and lactate levels in the first three hours after termination of glucose infusion. Lastly, deuterated water levels were relatively constant throughout the dynamic MRI measurement. Clearance profiles of the individual metabolites were not statistically different between groups.

### The cellular composition of stroke lesions differs between the subacute and chronic phases

**Figure 5.**
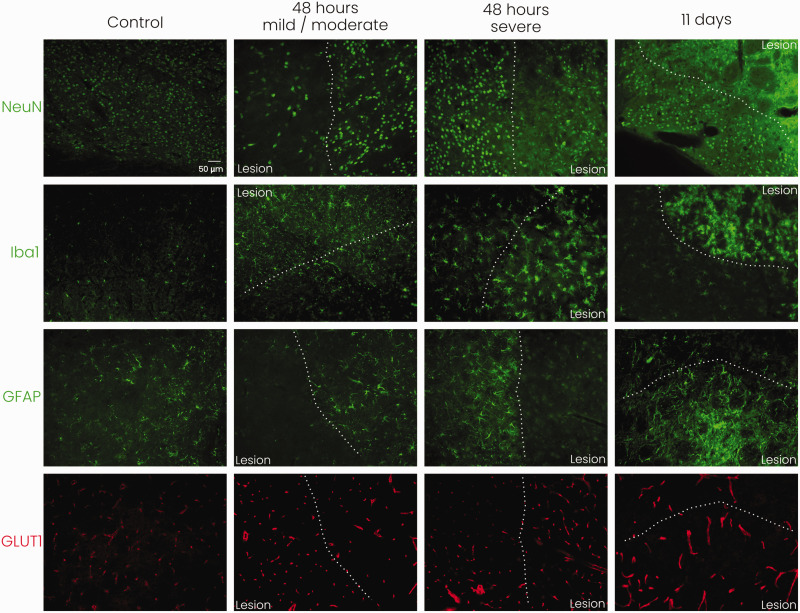
Representative immunohistochemical stainings for neurons (NeuN), microglia/macrophages (Iba1), astrocytes (GFAP), and microvessels (GLUT1) in mouse subcortical tissue (striatum) at the lesion border with 20× magnification. White dotted line indicates the lesion border. Staining reveals neuronal distress, microglial/macrophage activation, reactive astrogliosis, and increased signal of microvasculature as a consequence of stroke. NeuN: neuronal nuclear protein; Iba1: ionised calcium-binding adapter molecule 1; GFAP: glial fibrillary acidic protein; GLUT1: glucose transporter 1*; n = 1 for each experimental group, scale bar represents 50 μm and is similar for all images.*

We performed qualitative immunohistochemistry to assess the cellular status in post-stroke subcortical brain tissue (striatum). Staining for Iba1 (microglia/macrophages), GFAP (astrocytes), NeuN (neurons), and GLUT1 (microvasculature) are shown in Figure 5 (combined staining of Iba1, GFAP and NeuN with GLUT1 and DAPI are shown in Supplemental Figure 2). We observed enhanced Iba1 staining in and around the lesioned area at 48 hours and 11 days after tMCAO, with swollen cell bodies and altered morphology of the stained cells, showing activated microglia/macrophages. GFAP staining was enhanced in the lesion border at 48 hours showing increased ramification and size of GFAP-positive cells indicative of reactive gliosis. Decreased GFAP staining inside the lesion was observed after 48 hours, whereas at 11 days an enhancement of GFAP staining including increased ramification was observed in and around the lesioned area. NeuN staining of neuronal nuclei was reduced at both time-points after stroke, and a lower number of intact neurons was observed within the lesion. NeuN staining revealed somatic swelling, especially at 11 days after stroke, in and around the lesion. GLUT1 staining revealed enhanced signal (thickness, length) of microvasculature in the lesioned and perilesional areas at 48 hours and 11 days after stroke.

When comparing subcortical (striatal) tissue of mice with severe or mild/moderate stroke lesions, we observed more pronounced appearance of microglial cell bodies at 48 hours after severe stroke. Also, GFAP staining indicated a denser glial lesion barrier in mice with severe stroke. GLUT1 staining, neuronal swelling and loss of definition of neuronal nuclei (NeuN) appeared similar between severe and mild/moderate stroke injury.

## Discussion

In this study we applied FDG PET and DMI to assess changes in glucose metabolism in relation to lesion severity subacutely and chronically after cerebral ischemia-reperfusion in mice. FDG PET revealed reduced glucose uptake in the stroke-affected hemisphere at 48 hours after tMCAO, suggestive of metabolic depression despite reperfusion. This could not be explained by disrupted glucose delivery due to incomplete (micro)vascular reperfusion since DMI showed no differences in signal from infused deuterated glucose in the lesioned hemisphere as compared to healthy control brain. Similar observations of sustained post-stroke glucose metabolism depression, even after reperfusion, were described in landmark PET studies in patients and cats.^29–31^ DMI revealed active lactate formation, indicative of ongoing anaerobic metabolism, at 48 hours in the ipsilateral hemisphere. This was related to lesion severity, as significantly increased lactate formation was only measured in cortical and striatal ROIs within severe stroke lesions, and not in mild/moderate lesions. The measured higher levels of deuterated glucose compared to contralateral may have reflected hyperperfusion, which has previously been observed in rodent brain a few days after 60-minute tMCAO.^32^ At this stage, oxidative metabolism of the reperfused tissue, indicated by glutamate/glutamine synthesis, was significantly reduced in severe lesions, but not in mild/moderate stroke lesions. Oxidative metabolism remained reduced in cortical and striatal ROIs at post-stroke day 11 in severe lesions (no data available for mild/moderate stroke lesions), while lactate synthesis returned to baseline.

Immunohistochemistry showed neuronal loss, microglial/macrophage activation, and microvascular remodelling in the subacute lesions as well as reactive astrocytes in perilesional areas. Non-neuronal cell proliferation and vascular reorganisation inside the lesion territory and at the lesion border subacutely after tMCAO in mice has been reported previously.^33^ In the present study, microglial/macrophage activation was most pronounced in mice with severe stroke injury. The activity of microglia/macrophages, which are known to rapidly populate post-stroke lesioned tissue,^33^ could be responsible for the marked anaerobic metabolism, as these cells can produce lactate even under aerobic circumstances^34,35^ and prefer aerobic glycolysis during activation.^36^ Since the Iba1 staining does not distinguish between brain-resident microglia and blood-borne macrophages, the observed metabolic changes may also be (partly) caused by infiltrated immune cells. GFAP staining at 48 hours was particularly detected at the border of the lesion area, while barely visible in the lesion itself,^37^ suggesting that the elevated lactate formation did not specifically arise from activated astrocytes. However, due to partial volume effects, as a result of large MRSI voxels, lactate signal may have (partly) originated from perilesional reactive astrocytes.

Maintained glutamate/glutamine labelling in the mild/moderate lesions at 48 hours after tMCAO may reflect preserved neuronal and astrocyte glucose metabolism. However, our immunohistochemistry data showed significant loss of neurons, comparable to severely injured post-stroke tissue. We speculate that the preserved glutamate/glutamine metabolism with normal aerobic metabolism could arise from astrocytes and specific state-dependent microglia, as a recent study has shown that the metabolic state of microglia relies on the degree of proinflammatory stimulation.^38^ Microglial stimulation with lipopolysaccharide (LPS) combined with interferon-γ induced a high glycolytic and low aerobic phenotype, whereas a single LPS stimulus resulted in an energetic state characterised by an increase in lactate production while preserving aerobic metabolism. Thus, different degrees of proinflammatory stimulation of microglia may explain the observed differences in glucose metabolism in mild/moderate versus severe lesions subacutely after stroke.

Failed restoration of oxidative metabolism in the lesioned striatum and cortex in the chronic phase (severe stroke group) was further confirmed by our histological findings. Microglial/macrophage activation and astrogliosis were evident in the lesions after 11 days, while intact neurons remain sparse with evident neuronal swelling. Therefore, the recovery of glucose uptake measured with FDG PET at this stage most probably reflects glucose metabolised in glial and immune cells and is not a sign of neuronal recovery. Similarly, elevated lactate levels in infarcted tissue of chronic stroke patients, detected with ^1^H MRS, have been associated with macrophage activity.^1,39^ Interestingly, while Iba1 staining showed microglial activation already after 48 hours, we and others^40^ observed normalized [^18^F]FDG uptake levels only after a week or more after stroke. In apparent contrast with our findings, a consistent reduction of glucose consumption was observed in infarct and peri-infarct regions in patients who underwent PET scanning at 6–48 hours and 13–25 days after ischemic stroke.^41^ However, these patients did not receive recanalization therapy and perfusion levels remained lowered. In future studies, dynamic [^18^F]FDG measurements to calculate individual FDG net influx rate constants may further inform on actual glucose utilisation at different stages after cerebral ischemia-reperfusion.

We hypothesised altered metabolite clearance based on previously reported dysfunction of the glymphatic system after stroke.^15,16^ Our dynamic DMI experiment after infusion of the deuterated glucose enabled measurement of metabolite clearance in the (post-stroke) mouse brain. This pilot substudy did not reveal significant changes in metabolite clearance profiles as a result of stroke. Whether this is due to the small sample size, the timing of the measurement or the subtlety of the changes rendering them undetectable with DMI remains to be elucidated.

Our study has some limitations. First, the voxel size of the DMI data was relatively large compared to the small mouse brain. This is inherent to MRSI where voxels are larger than for MRI, which increases susceptibility for partial volume effects. We mitigated these partial volume effects by excluding voxels with insufficient SNR due to signals from outside the brain. Additionally, by artificially increasing the MRSI resolution, relative signal contribution for metabolite quantifications could be adjusted based on the degree of overlap with the MRI-based ROIs. Second, because of the inherent variability of the tMCAO stroke model,^25^ mild/moderate stroke injuries were not evenly represented in the subacute and chronic stroke groups. This could not be corrected for, because we used a cross-sectional study design, in which we were blinded to stroke severity until the final imaging time point. Third, we did not perform neurological scoring of the mice, which would have allowed assessment of the relation between our imaging findings and functional outcome. Fourth, no serial imaging was performed in this study. Longitudinal study designs require careful evaluation of the clearance profiles of the tracers to prevent confounding factors and therefore it was chosen to perform a cross-sectional study in our exploratory study. Additionally, the PET and MRI experiments were carried out in different facilities with different animals, precluding direct cross-modality analyses. Fifth, an ROI inside the MCA territory was manually outlined on CT scans as we did not apply a contrast agent for lesion delineation. Consequently, we could not perform specific FDG PET analysis of lesioned cortical and striatal tissue. Sixth, due to the exploratory nature of our study, the sample sizes were relatively small. The histopathological studies were based on single samples and only analysed qualitatively. Further analyses are required to fully elucidate the underlying pathological mechanisms of metabolic changes in post-stroke brain.

In this study we combined complementary metabolic imaging techniques providing multifaceted information on the metabolic status of post-stroke tissue. FDG PET has been widely applied as a method for diagnosis and monitoring of treatment responses. As demonstrated by us, the clinically applicable and non-invasive imaging method DMI can further reveal distinct metabolic profiles in post-ischemic tissue, which depend on the level of stroke severity. Our findings show that stroke severity modulates metabolic changes in the brain and that DMI may inform on the underlying status of the ischemic tissue, including neuronal distress, glial activation, and metabolic changes due to inflammatory processes.

## Supplemental Material

sj-pdf-1-jcb-10.1177_0271678X221148970 - Supplemental material for *In vivo* imaging of cerebral glucose metabolism informs on subacute to chronic post-stroke tissue status – A pilot study combining PET and deuterium metabolic imaging Click here for additional data file. Supplemental material, sj-pdf-1-jcb-10.1177_0271678X221148970 for *In vivo* imaging of cerebral glucose metabolism informs on subacute to chronic post-stroke tissue status – A pilot study combining PET and deuterium metabolic imaging by Anu E Meerwaldt, Milou Straathof, Wija Oosterveld, Caroline L van Heijningen, Mandy MT van Leent, Yohana C Toner, Jazz Munitz, Abraham JP Teunissen, Charlotte C Daemen, Annette van der Toorn, Gerard van Vliet, Geralda AF van Tilborg, Henk M De Feyter, Robin A de Graaf, Elly M Hol, Willem JM Mulder and Rick M Dijkhuizen in Journal of Cerebral Blood Flow & Metabolism
