# Supplementary material for: In vivo imaging of cerebral glucose metabolism informs on subacute to chronic post-stroke tissue status – A pilot study combining PET and deuterium metabolic imaging
Source: J Cereb Blood Flow Metab. 2023 Jan 6;43(5):778–90. doi: 10.1177/0271678X221148970 (PMC10108187; doi:10.1177/0271678X221148970)

Supplemental Material

Supplemental Figure 1. Concentrations of deuterated water, glucose and its deuterated metabolites in ipsi- and contralateral mouse brain (control, and 48 hours and 11 days post-stroke) during the first dynamic DMI clearance measurement after stopping deuterated glucose infusion. There were no significant differences between groups in the water, glucose and metabolite concentrations at the start of the dynamic clearance measurement.. *I = ipsilateral, C = contralateral, n = 2 for control, n = 9 for 48 hours post-stroke, n = 4 for 11 days post-stroke, mean±SD.*

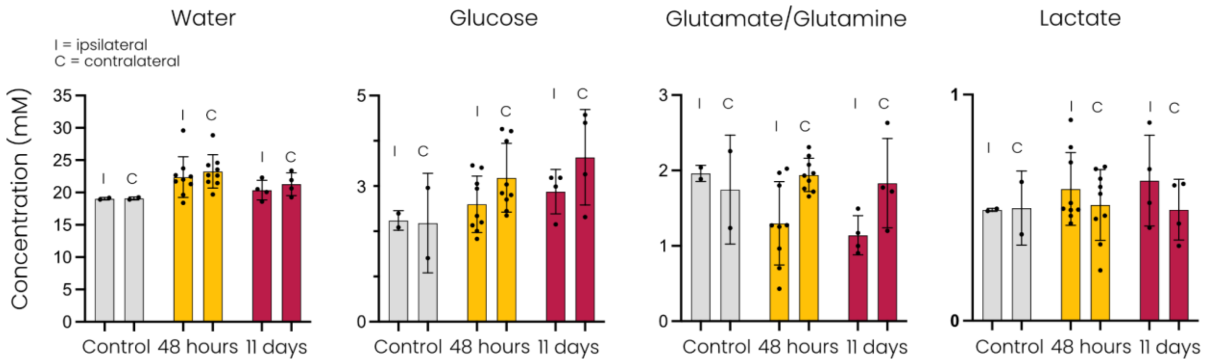

Supplemental Figure 2. Representative composite images of immunohistochemical NeuN/Iba1/GFAP-GLUT1-DAPI staining in mouse subcortical tissue at the lesion border with 20x magnification of a control mouse, mice with severe or mild/moderate injury 48 hours after tMCAO, and a mouse at 11 days after tMCAO. White arrows indicate the lesion border with the arrowhead pointing towards the lesion. *NeuN (green) = neuronal nuclear protein, Iba1 (green) = ionized calcium-binding adapter molecule 1, GFAP (green) = Glial fibrillary acidic protein, GLUT1 (red) = Glucose transporter 1, DAPI (blue) = 4',6-diamidino-2-phenylindole; n = 1 for each experimental group, scale bar represents 50 µm and is similar for all images.*

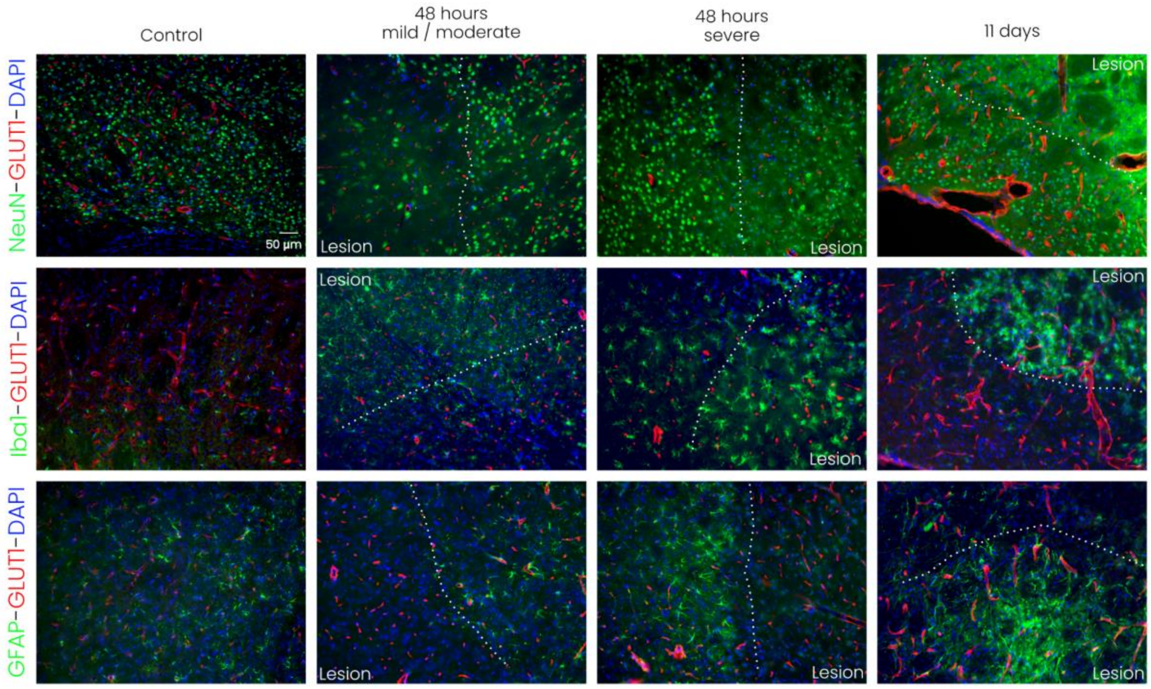

Supplement: sj-pdf-1-jcb-10.1177_0271678X221148970 - Supplemental material for In vivo imaging of cerebral glucose metabolism informs on subacute to chronic post-stroke tissue status – A pilot study combining PET and deuterium metabolic imaging [file sj-pdf-1-jcb-10.1177_0271678X221148970.pdf]
